# Influence of electrical and thermal properties on RF ablation of breast cancer: is the tumour preferentially heated?

**DOI:** 10.1186/1475-925X-4-41

**Published:** 2005-07-11

**Authors:** Vilhelm Ekstrand, Hans Wiksell, Inkeri Schultz, Bengt Sandstedt, Samuel Rotstein, Anders Eriksson

**Affiliations:** 1 Department of Surgical Sciences, Karolinska Institutet, Stockholm, Sweden; 2 VibraTech AB, Stockholm, Sweden; 3 Department of Surgery, Karolinska Institutet at Danderyd's Hospital, Stockholm, Sweden; 4 Department of Pathology, Karolinska Institutet at Danderyd's Hospital, Stockholm, Sweden; 5 Department of Oncology, Karolinska University Hospital, Stockholm, Sweden; 6 Department of Mechanics, Royal Institute of Technology, Stockholm, Sweden

## Abstract

**Background:**

Techniques based on radio frequency (RF) energy have many applications in medicine, in particular tumour ablation. Today, mammography screening detects many breast cancers at an early stage, facilitating treatment by minimally invasive techniques such as radio frequency ablation (RFA). The breast cancer is mostly surrounded by fat, which during RFA-treatment could result in preferential heating of the tumour due to the substantial differences in electrical parameters. The object of this study was to investigate if this preferential heating existed during experimental in vitro protocols and during computer simulations.

**Methods:**

Excised breast material from four patients with morphologically diagnosed breast cancers were treated with our newly developed RFA equipment. Subsequently, two finite element method (FEM) models were developed; one with only fat and one with fat and an incorporated breast cancer of varying size. The FEM models were solved using temperature dependent electrical conductivity versus constant conductivity, and transient versus steady-state analyses.

**Results:**

Our experimental study performed on excised breast tissue showed a preferential heating of the tumour, even if associated with long tumour strands. The fat between these tumour strands was surprisingly unaffected. Furthermore, the computer simulations demonstrated that the difference in electrical and thermal parameters between fat and tumour tissue can cause preferential heating of the tumour. The specific absorption rate (SAR) distribution changed significantly when a tumour was present in fatty tissue. The degree of preferential heating depended on tissue properties, tumour shape, and placement relative to the electrode. Temperature dependent electrical conductivity increased the thermal lesion volume, but did not change the preferential heating. Transient solutions decreased the thermal lesion volume but increased the preferential heating of the tumour.

**Conclusion:**

Both the computer model and the in vitro study confirmed that preferential heating of the tumour during RFA exists in breast tissue. However, the observed preferential heating in the in vitro studies were more pronounced, indicating that additional effects other than the difference in tissue parameters might be involved. The existing septa layers between the cancer tissue and the fatty tissue could have an additional electrical or thermal insulating effect, explaining the discrepancy between the in vitro study and the computer model.

## Background

At least 10% of the women in the western world face the prospect of developing breast cancer. The tendency in modern treatment of these tumours is towards less invasive local treatment. Today breast conserving surgery (BCS) has become more common than mastectomy in many countries. BCS and mastectomy combined with radiation are associated with satisfactory long-term outcome. The survival rates after BCS of ductal carcinoma in situ is approximately 98%, whereas approximately 100% of these patents are cancer free after mastectomy [1,2]. However, multiple treatments and additional adjuvant care are needed in up to 50% of the BCS cases, resulting in higher associated costs compared to mastectomy alone [3,4]. As in all surgery for breast cancer, the goal is to remove all of the cancertogether with a sufficient margin of healthy tissue, to prevent local recurrence. Furthermore, today many breast cancers are detected at an early stage by mammography screening, raising a demand for new techniques that minimise alternation of breast configuration. Recently, approaches other than traditional surgery have been explored to satisfy these demands [5-7]. These techniques are minimally or totally non invasive, and include, cryosurgery, stereotactic excision, laser ablation, focused ultrasound, and radio frequency ablation (RFA). Potential benefits with these techniques are reduced morbidity rates, reduced treatment duration, and the ability to perform therapy for patients in poor medical condition on an outpatient basis. Of these new techniques, RFA is considered to be the most promising treatment for breast cancer because of its effective destruction of cancer cells and having a low complication rate [8,9].

**Figure 1:**
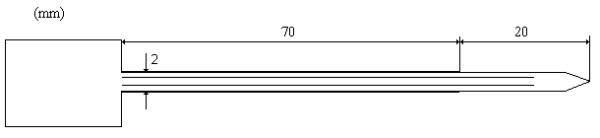
**Treatment electrode**. Outline of the treatment electrode with incorporated cooling lumina and electrical isolation layer.

RFA devises induce thermal tissue necrosis in the target region. Temperature elevation is caused by ion agitation, which is converted into heat by the effect of friction. Current is generated by an applied voltage between two electrodes. For the monopolar regime, these are the non-isolated part of a treatment (active) electrode (see figure 1), and an indifferent (passive) electrode. The active part of the electrode is introduced into the tumour with ultrasonic guidance. The current flowing from the active part heats the surrounding tissue. In the frequency range used (1.5 MHz), the so called antenna current due to external radiation is fairly low, i.e. the electrodes carry an almost fully balanced current that flows in pathways of least resistance between the electrodes. During RFA activation, the imaginary part of the energy is negligible. Consequently, henceforth we will use only the real-part of the admittance (i.e. conductivity) in the equations describing the process.

The absorbed power density at each point is:



where q = power density (W/m^3^), E = electric field (V/m), J = current density (A/m^2^) and σ = conductivity (1/(Ω·m)) (Vector variables have both a magnitude and a direction and are presented with an over-bar.). The total current through each closed surface enclosing one of the electrodes always equals the total current from the generator, independent of surface size. Hence, the heating will be concentrated in the region close to the active electrode, where the surface area is small and the current density is high. During experimental protocols with our electrode in in vitro muscle tissue, 87% of the total electrical energy is absorbed in an iso-potential volume with radius 2 cm [10]. Between 43°C and 60°C the cell damage originates from denaturation of proteins. Over 60°C the tissue coagulates, because collagen is converted to glucose and the time frame of cell death is considered almost instantaneous. When the tissue temperature reaches approximately 90–110°C, phase transformation of the intra- and extra-cellular liquids occurs. Glucose develops an adhesive effect after dehydration. The gas bubbles created function as electrical insulation, which alters the effective current-path area and further increases the current density and tissue temperature. If the temperature reaches 200°C, tissue charring is initiated. This avalanche-like phenomenon ultimately insulates the electrode from the tissue, and heating often ceases non-reversibly. Thus, the output power during RFA is limited, ensuring that the maximum tissue temperature is below the initiation of the avalanche-like insulation phenomenon. The coagulation necrosis zone with one monopolar electrode will therefore be limited to approximately 13 mm in diameter during in-vivo protocols in muscle tissue [7]. Several approaches have been proposed to increase the necrotic region. These are multiprobe electrodes [11], saline injected electrodes [12], and internally cooled electrodes [13]. Ultrasonography, which is used to guide the electrode to the tumour, cannot adequately predict the thermal lesion margin. Instead, MRI and/or a core biopsy can be used to confirm adequate ablation [14]. The success rate of the in-vivo studies performed before mastectomy varyies from 86% (19–22) to 100% (21–21) [14-17]. This variation might depend on different inclusion criteria, e.g. tumour size. The procedure was well tolerated, without complications and cosmesis was excellent. To date, no studies have used RFA as the only treatment method for breast cancer. Hence, the long-term oncologic results of this new method are yet to be evaluated.

The normal female breast is composed primarily of fat with varying concentration partly due to body habitus. The breast gland is composed of lobes, which empty into separate major ducts terminating in the nipple. Each lobe and its smaller subunits are separated by connective tissue. The amount of the latter usually increases with age. There are two types of breast cancers, which constitute more than 95% of the malignant tumours; ductal and lobular carcinomas. The former is derived from duct and the latter from terminal duct epithelium. They differ greatly in morphological as well as biological aspects. The classical "crablike" appearance of the ductal carcinoma, with solid central body from which strands stretch out in the surrounding tissue has, in fact, given name to all cancers (Krebs in German). The radiating branches consist of tumour cells and connective tissue. The latter partly considered to be induced by the tumour. The lobular carcinoma, on the other hand, has a diffuse growth pattern, is often multifocal, and is difficult to diagnose radiologically. Thus, RFA treatment of breast cancer comprises a unique situation, since the tumour is mostly embedded in fat. The difference in electrical parameters between the fat and the tumour tissues is substantial, which might result in preferential heating of the tumour. The goal of this study was to document if preferential heating existed during in vitro treatment, and if it could be induced in computer simulation by the dissimilarity of electrical and thermal parameters.

Several computer simulation studies have investigated RFA in a variety of locations such as the liver [18-21] or the heart [22]. The main objective in most of these studies was to predict the thermal lesion size. Other relevant issues such as the effects of electrode cooling [21], vessel size [20], multiple probes [19], and temperature dependent conductivity [18] have also been addressed. Earlier FEM studies on breasts have primarily studied thermography for diagnostic purposes [23,24].

## Methods

### Experimental In vitro study

The in vitro study, approved by the local ethics committee, was performed on excised breast material with a morphology diagnosis of breast cancer. After obtaining informed consent, four patients underwent modified radical mastectomy. After surgery the specimens, three ductal and one lobular carcinomas all over three centimetres in diameter, were sent for pathologic examination. Subsequently, an internally cooled steel electrode (VibraTech AB, Stockholm, Sweden, figure 1) was installed in the central part of the tumour. Temperature was measured at the needle tip with incorporated thermo-couples. The RF-generator was specially designed with a floating low-impedance output (0–950 W, 1.5 MHz). The maximum tissue temperature was maintained at approximately 100°C over 15 min. It is difficult to measure the true maximum temperature due to the circulating cooling media, i.e. the true maximum is located within the tissue. We have developed an algorithm that compensates for the deviation between the maximum temperature and the measured value at the electrode tip. Electrical impedance was measured to ensure that the initiation of the avalanche phenomenon did not occur. The temperature and the flow of the cooling media were 20°C and 12 ml/s, respectively. An electrode current (root mean squared) between 0.35 and 0.71 A was applied during the procedure. After the treatment, the tissue was immersed and fixed in a 4% formalin solution. The tumour, including the electrode canal and surrounding fat tissue, was cut out, trimmed, embedded and processed for large sections, which underwent histologic examination. Thermal lesion margin was defined as the zone with well established coagulation necrosis, i.e. condensation and loss of nuclear details and homogenisation of the cytoplasm.

### Computer simulation

#### Governing equations

Because the tissue heating is induced by RF-energy, a quasi-static electrical model can be used to describe the electrical field and the current density. Under quasi-static conditions the electric potential can be solved using Laplace's equation:

▽·[σ(*T*)·▽*V*] = 0    (2)

where V is the electrical potential (V) and T is the temperature (°C). The thermal behaviour is governed by the bio-heat equation:



where ρ is the tissue density (kg/m^3^), c is the heat capacity (J/kg°C), k is the thermal conductivity (W/°C m), q is the electrical energy source, ρ_b _is the density of blood, C_b _is the heat capacity of blood, ω is the blood perfusion, T_blood _is the basal temperature of the blood, and Q_m _is the metabolic heat source. In an in-vitro situation, the metabolic heat source and the blood perfusion are set to zero.

#### Geometry

**Figure 2:**
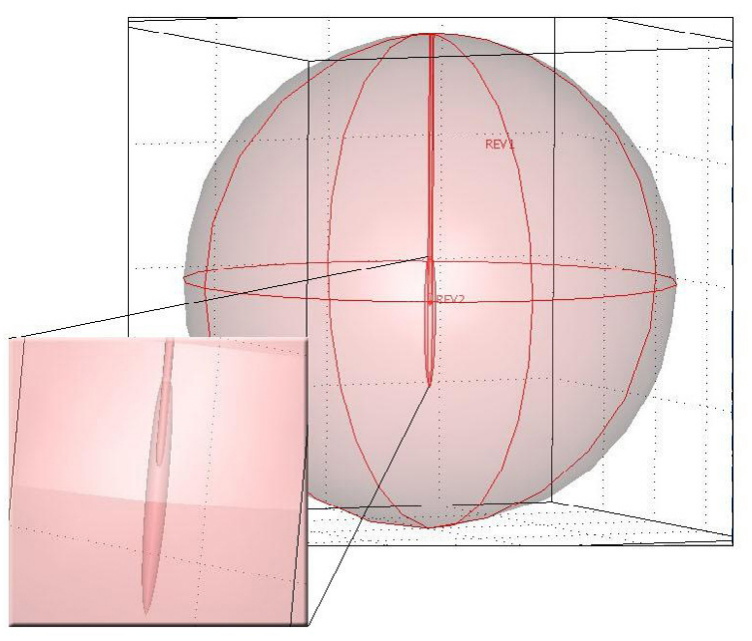
**Model geometry with tumour**. The model geometry consists of two tissue elements, fat and tumour. In some cases the tumour is not included in the model. The geometry is rotationally symmetric, where the diagonal symmetry axis is centred in the horizontal plane. All model geometries consist of a spherical isotropic volume of fat (d = 200 mm). Additionally, in most cases, a smaller cancerous tissue mass is added into the model along the symmetry axis. The tumour dimensions in this figure are 52 mm × 4.7 mm. The electrode is incorporated in the model by removing tissue with the same geometry as the electrode and setting appropriate boundary conditions at the electrode-tissue interface. The tissue-electrode boundary is positioned around the symmetry axis, where the active part is placed in the middle of the spherical volume. In the enlarged section, we see that the tumour is placed around the electrode along the symmetry axis, with 63% of its length below the electrode.

**Figure 3:**
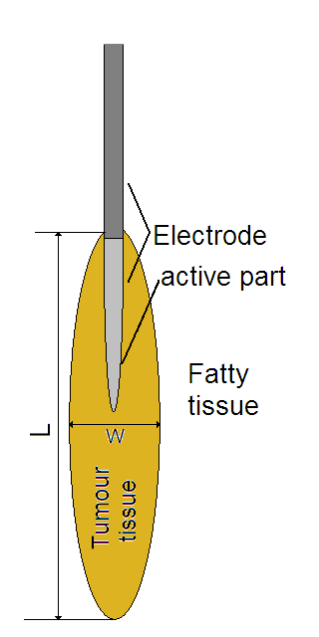
**Model geometry close to the electrode**. W = width and L = length of the tumour.

Because of the need to save computer resources, the geometry of the computer model was defined using rotational symmetry with cylindrical coordinates. The axial-symmetric assumption requires axial-symmetry in geometry, materials, loadings, and boundaries, which therefore also leads to an axial-symmetric solution. All model geometries consist of a spherical isotropic volume of fat (d = 200 mm) and a tissue-electrode boundary. In most cases tumour tissue is also included in the model, figure 2 and figure 3. The electrode have the same dimensions as the one used in the experimental studies and is incorporated in the model by setting appropriate thermal and electrical boundary conditions to the tissue-electrode interface. The tissue-electrode boundary is positioned around the symmetry axis, where the active part is placed in the middle of the spherical volume. In cases 1, 2 and 9, only fatty tissue is used in the model (table 1). The remaining cases have additional cigar shaped cancerous tissue, with variable length (L) and width (W), positioned around the electrode along the symmetry axis (figure 3). The majority (63%) of the cancer length is located below the electrode tip. The shape and the placement of the tumour model should give a similar preferential effect to the tumour strands extending from the tumour. The tumour boundary consists of a rotated third degree Bezier curve, to avoid sharp edges along the tumour-fat interface that could distort the specific absorption rate (SAR) distribution. Bezier curves create smooth curves by linearly interpolating the slope between points.

**Table 1:** Description of cases

Case description		Tumour dimensions (mm)		**C**onst / **T**emp dep. σ	Number of Elements	**S**teady state/**C**ontrolled **T**ransient
					
		Width	Length			
1	F	-	-	C	1921	S
2	F	-	-	T	5520	S
3	FT	4.7	52	C	2208	S
4	FT	4.7	52	T	2214	S
5	FT	4.7	104	T	2797	S
6	FT	4.7	26	T	1940	S
7	FT	9.4	52	T	1985	S
8	FT	2.35	52	T	1985	S
9	F	-	-	T	5230	CT
10	FT	4.7	52	T	2214	CT

F = geometry with only fatty tissue, FT = geometry with fatty tissue and embedded tumour. C and T indicate constant and temperature dependent electrical conductivity respectively. S indicates that steady-state analysis has been performed, i.e. when the model has reached thermal equilibrium using constant boundary conditions. Cases denoted CT use transient analysis where Vin is controlled to maintain the maximum temperature at 100°C.

#### Tissue parameters

Several previous studies have investigated the thermal and electrical parameters of breast tissue [24-29]. The electrical properties used in our FEM model have been measured by Jossinet [29] for electrical impedance tomography (EIT) applications (table 2). Jossinet has done the most extensive study, which includes in vitro measurements from 64 patients. Thermal conductivities were gathered by Gautherie, et al. [25], which is the most commonly used data. As indicated by T in table 1, in case 2 and 4–10, temperature dependent electrical conductivity was used. The temperature dependence of electrical conductivity in most ion solutions and tissues is 2%/°C [30,31]. Whenever temperature dependent electrical conductivity is included in the model, this relationship is used.

**Table 2:** Electrical and Thermal parameters

**Tissue type**	**Electric Conductivity (1/(Ω·m))**	**Thermal Conductivity (W/(m·°C))**	**Specific heat (kJ/(kg·°C))**	**Density (kg/m^3^)**
**Fat**	0.057^[29]^	0.12^[25]^	3^[27],[26]^	920^[27]^
**Tumour (carcinoma)**	0.412^[29]^	0.28^[25]^	3.5^[26]^	1000^[26]^

The electrical parameters are measured at 1 MHz.

#### Boundary conditions

The electrical boundary condition for the outer boundary was set to V = 0, representing the ground electrode. The electrical boundary condition for the conducting electrode was set to a source potential of V = V_in_. The non-conducting part had an insulating boundary condition, i.e. the current component orthogonal to the surface is zero. The thermal boundary condition of the outer surface was set to 20°C, i.e. the initial temperature of the tissue. With our equipment, the temperature at the electrode boundary does not diverge far from the cooling media temperature. Thus, the thermal boundary condition of the electrode was set to the cooling media temperature, T = 20°C, to incorporate the electrode cooling in the model.

#### Numerical model

Ten finite element method (FEM) [32,33] models were developed (cases 1–10, table 1), and solved using FEMLAB 3.0 (Comsol, Stockholm, Sweden) on a 2.0 GHz AMD Athlon XP 2400 computer, with 1024 Mb RAM. When the electrical conductivity is independent of temperature, the electrical potential, V, can be found without solving the bio-heat equation. Thus, the calculated V, defined over the entire volume, is subsequently used to solve the bio-heat equation. When the electrical conductivity is temperature dependent, equations 2 and 3 must be solved by a coupled method. The required iterative computations for these non-linear problems are much more computer intensive. All models were solved without including blood perfusion and metabolic heating. Cases 1–8 were solved using a steady state approximation, where V_in _was adjusted to obtain a final maximum temperature of 100°C. Additionally, two controlled 15 min transient simulations were performed, cases 9 and 10. During these simulations, the maximum temperature was maintained at approximately 100°C throughout the whole treatment, by successively adapting the boundary condition V_in_.

A quadratic mesh consisting of Lagrange triangular elements was used for both the thermal and the electrical problems. The conservationof energy introduced by the source was checked and found highly reliable. This measurement serves as an indication of accuracy in the formulation. Furthermore, a common ad hoc procedure of successive mesh refinement was used, with the FEM solution considered converged when the difference in maximum temperature between successive calculations was less then 0.1% for a doubling of the number of elements. Thermal lesion size was determined using the 50°C margin. Even though the thermal lesion volume is dependent on both temperature and time of elevated temperature, similar lesion dimensions have been obtained using both thermal dose and threshold temperature [34].

## Results

### Experimental in vitro study

**Figure 4:**
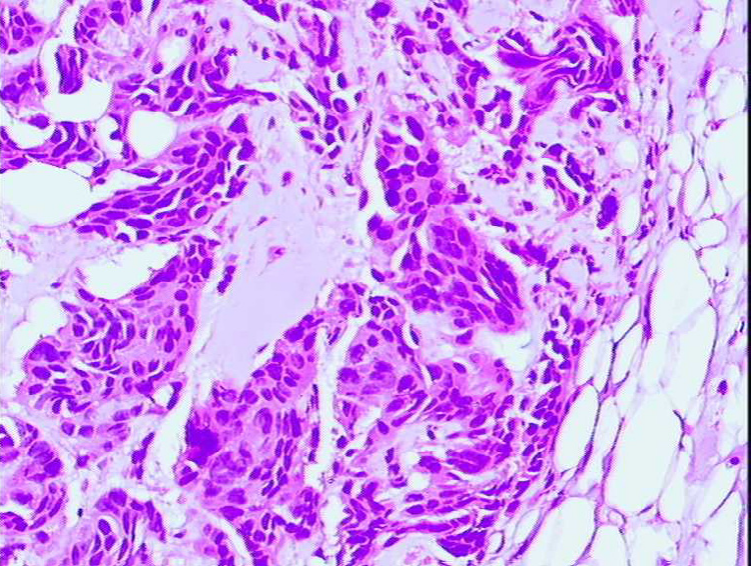
Ductal carcinoma cells with early coagulation necrosis. H&E × 250.

**Figure 5:**
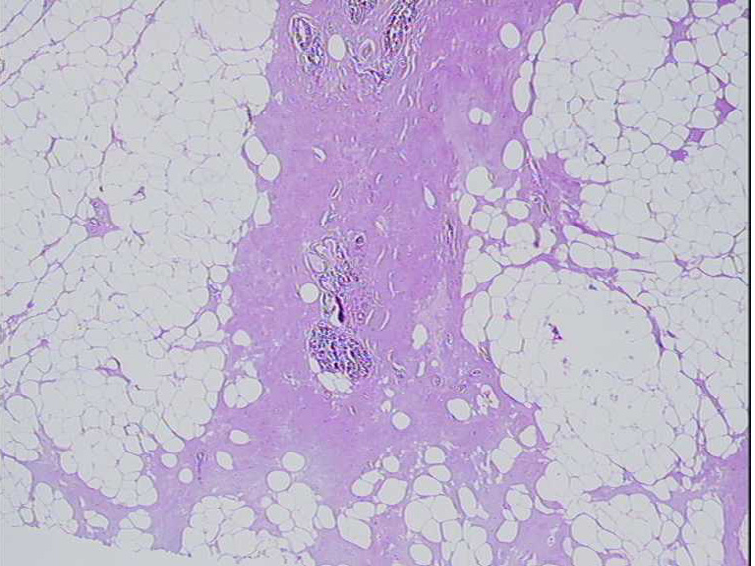
Tumour strand with acellular degenerated connective tissue and non viable intraductal carcinom. H&E × 100.

In a circular area around the electrode canal there was a well established coagulation necrosis, i.e. condensation and loss of nuclear details and homogenisation of the cytoplasm (figure 4). The thermal lesion diameter was approximately 2–3 cm. The changes were evident in the tumour epithelium, connective tissue, and a rim of adjacent fat tissue, but were also seen in the fibro-tumourous strands up to 3–8 mm from the main tumour mass (figure 5). However, the fat tissue between these ramifications, within a thin well-delineated rim, approximately 1 mm, showed only slight vacuolar degeneration and preserved nuclei, but was otherwise surprisingly unaffected. The ductal carcinomas showed no areas with viable tumour. In contrast, the lobular carcinoma with its irregular outline and diffuse growth showed tumour cells with preserved viable appearance in the surrounding fat at a distance from the coagulated area around the electrode canal. The impedance decreased up to 50% during the treatment.

### Computer simulation

**Figure 6:**
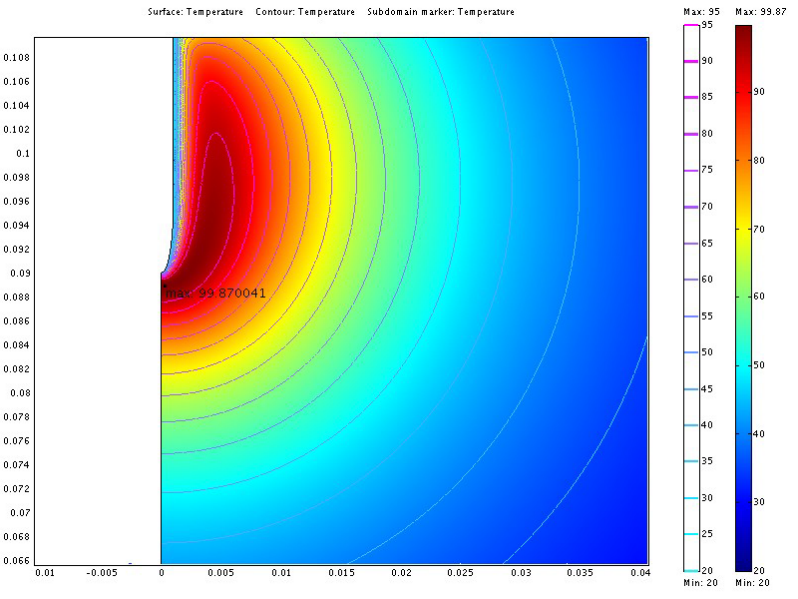
**Temperature distribution from case 2**. The illustration shows half the plane through the symmetry axis. The coordinates are cylindrical where the horizontal axis is denoted r and the vertical axis z. The symmetry axis located at r = 0. The redundant three dimensional solution is obtained by rotating the half plane about the symmetry axis. The electrode is located along the symmetry axis where Z > 0.09. The length scale of the axes is in meters. Isothermal lines from 20 to 95°C in steps of 5°C are also shown. The temperature distribution shows the formation of a spherically shaped thermal lesion around the electrode. The cooling of the electrode shifts the temperature maximum away from the electrode.

**Figure 7:**
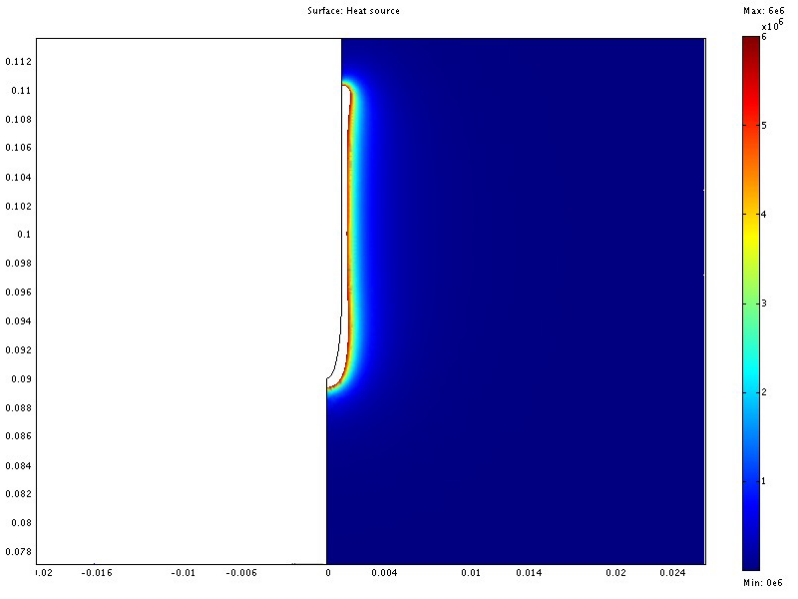
**Electrical absorption from case 2**. The illustration shows half the plane through the symmetry axis. The coordinates are cylindrical where the horizontal axis is denoted r and the vertical axis z. The symmetry axis located at r = 0. The redundant three dimensional solution is obtained by rotating the half plane about the symmetry axis. The electrode is located along the symmetry axis where Z > 0.09. The length scale of the axes is in meters. The absorption of electrical energy is almost evenly distributed along the electrode. Higher absorption occurs at the tip and the endpoint of the electrode isolation.

**Figure 8:**
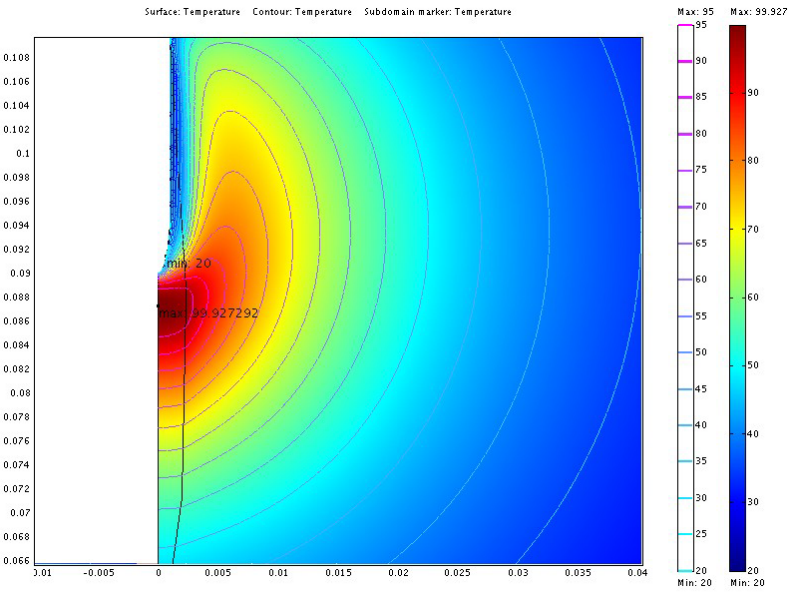
**Temperature distribution from case 6**. The illustration shows half the plane through the symmetry axis. The coordinates are cylindrical where the horizontal axis is denoted r and the vertical axis z. The symmetry axis located at r = 0. The redundant three dimensional solution is obtained by rotating the half plane about the symmetry axis. The electrode is located along the symmetry axis where Z > 0.09. The length scale of the axes is in meters. Isothermal lines from 20 to 95°C in steps of 5°C are also shown. The rough shape of the tumour is misleading because the Bezier curves are represented by a finite number of line segments. The tumour shifts the heat from the electrode downwards along the tumour. Consequently, the 95°C isotherm is now located below the electrode tip.

**Figure 9:**
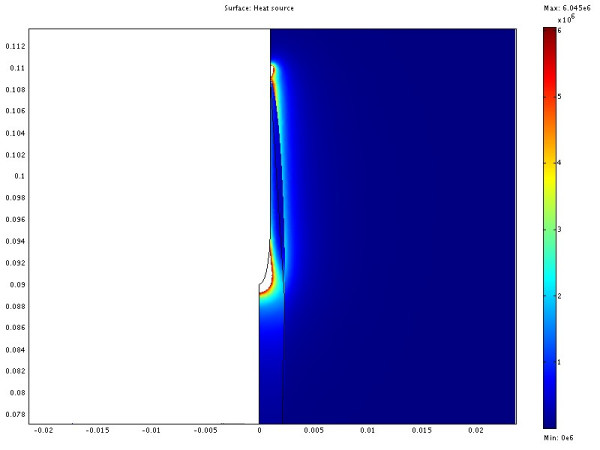
**Electrical absorption from case 6**. The illustration shows half the plane through the symmetry axis. The coordinates are cylindrical where the horizontal axis is denoted r and the vertical axis z. The symmetry axis located at r = 0. The redundant three dimensional solution is obtained by rotating the half plane about the symmetry axis. The electrode is located along the symmetry axis where Z > 0.09. The length scale of the axes is in meters. The tumour shifts the absorption of electrical energy to the tumour area and to some extent the tumour fat interface. The absorption in the tumour is always higher compared to the fat at the same distance from the electrode.

**Figure 10:**
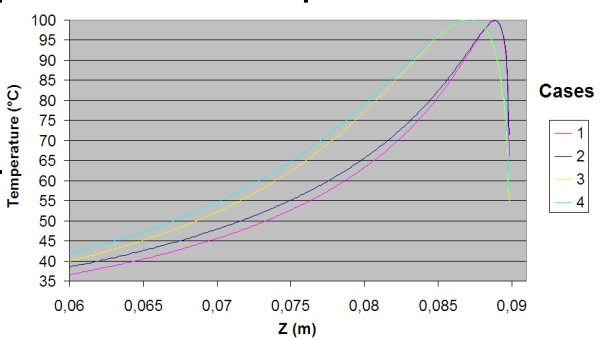
**Temperature distribution along the symmetry axis r = 0 for cases 1 to 4**. Both temperature dependent and constant conductivity was used for the only fat models and the tumour models. The electrode tip was located at Z = 0.09. Temperature dependent conductivity creates significantly bigger thermal lesions. Additionally, the tumour increases the temperature in the tumour compared to the models with only fat. The 50°C margin is increased 4.7 mm in the tumour direction for both temperature dependent and constant conductivity when the tumour is incorporated, i.e. the difference in thermal lesion size between the tumour and non-tumour models is not affected by the used conductivity type.

**Figure 11:**
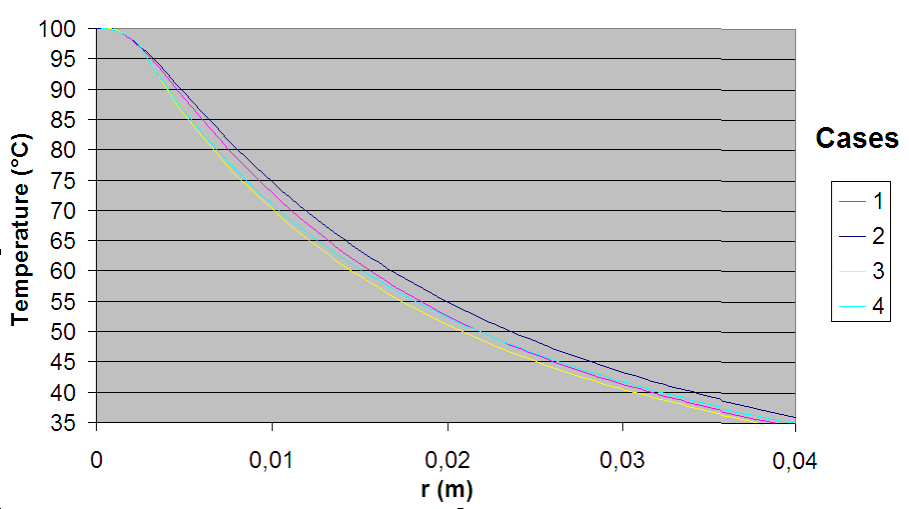
**Temperature distribution along the Z = constant line through Tmax for cases 1 to 4**. For case 1, 2 and 3, 4 this line was through Z = 0.087 and Z = 0.0889 respectively. Both temperature dependent and constant conductivity was used for the tumour models and the homogenous non-tumour models. Temperature dependent conductivity has the same effect on thermal lesion size as in the previous figure. Interestingly the tumour cases resulted in lower temperatures in this direction compared to the only fat cases.

**Figure 12:**
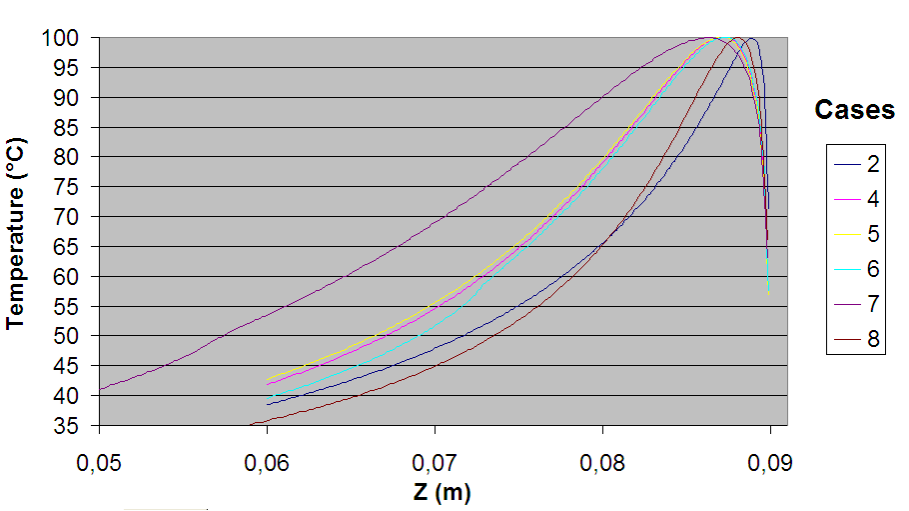
**Differences in temperature distribution along the symmetry axis for tumour models of variable size**. Tumour models with varying length and thickness are compared to the only fat case 2. Only temperature dependent conductivity was used. The electrode tip was located at Z = 0.09. The only spatial variable that had any significant effect on the temperature distribution was the tumour width W. Increased width enlarged the thermal lesion and pushed the maximum temperature further from the electrode. Interestingly, very thin tumours generated smaller thermal lesions (50° margin) than the original fat model.

**Figure 13:**
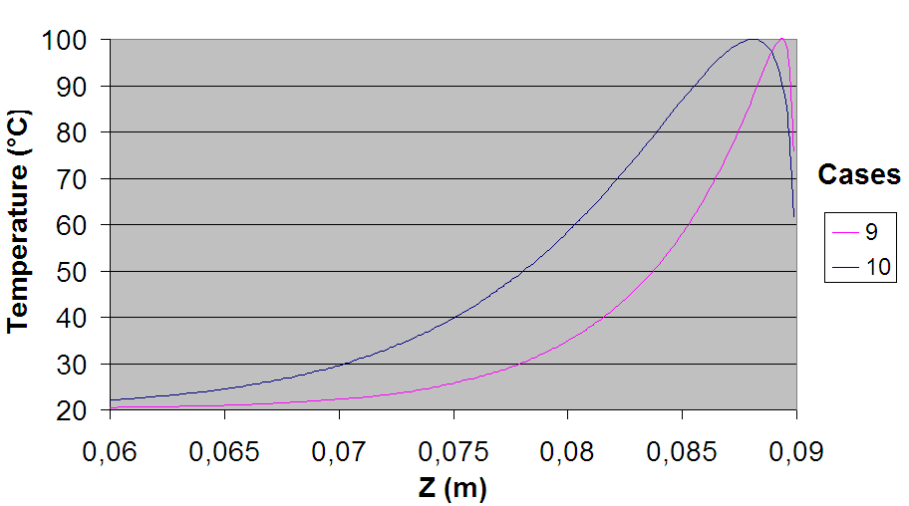
**Temperature distribution along the symmetry axis for the transient controlled simulations**. Two controlled transient solutions were compared, one only fat model (case 9) and one tumour model with tumour size 52 mm × 4.7 mm (case 10). T_max _was kept at 100°C during the whole 15 min treatment using temperature dependent conductivity in both cases. These simulations resulted in significantly smaller lesion compared to their steady-state counterpart. However, the difference in thermal lesion between the tumour and the non-tumour case increased.

The power consumption, impedance, voltage and current during the treatment for each of the cases are shown in table 3. The resulting temperature distribution from simulation with only fat tissue (case 2) and fat with embedded cancer (case 4), using temperature dependent electrical conductivity, is shown in figure 6 and 8 respectively. The maximum temperature in case 2 is located on the symmetry axis, 1.1 mm from the electrode tip. In case 4, where a tumour is situated in the fat, the heat pattern is drawn further from the tip along the tumour. The maximum temperature is located on the symmetry axis, 2.9 mm from the electrode tip. The difference in electrical power absorption between the cases with and without tumour is substantial (figures 7 and 9). When comparing solutions computed with constant and temperature dependent electrical conductivity, and with and without tumours along the symmetry axis, r = 0 (cases 1–4), the resulting thermal lesions were largest using temperature dependent electrical conductivity (figure 10). Along a line with constant Z-coordinate, through the maximum temperature point, the same observation can be made (figure 11). The smallest thermal lesions were created in case 1 (1.7 cm from the electrode) and case 3 (2.2 cm), in the r = 0 and the Z = const direction, respectively. The 50°C lesion margin was approximately 1–2 mm further from the electrode when temperature dependent electrical conductivity was used in the simulation. When tumour tissue was added to the fat model (cases 3 and 4), the 50°C margin increased by approximately 4.7 mm in the tumour direction (r = 0) and decreased by approximately 1.5 mm along the Z = const. line, through T_max_, using both temperature dependent and constant electrical conductivity. The temperature plot along the symmetry axis (r = 0) for different tumour dimensions (cases 4–8) using temperature dependent electrical conductivity can be observed in figure 12. The temperature profile does not substantially change when altering the length of the tumour. However, a small drop in case 6 is observed at the tumour-fat interface. The width of the tumour, on the other hand, has a considerable effect on the temperature along the tumour. Both the thermal lesion margin and the location of the temperature maximum were located further from the electrode with increasing tumour width. Surprisingly, the 50°C margin in case 8 was closer to the electrode than in case 2. The tissue impedance decreased by approximately 41–46% during the temperature dependent simulations. Transient controlled simulations created smaller thermal lesions than their steady state counterpart (figure 13). The shift in the 50°C margin between the tumour and non-tumour transient cases (9 and 10) was 5.6 mm along the symmetry axis. During the initial time period, the isothermal lines were clearly deformed in the tumour direction. However, this shape gradually disappeared and became more and more spherical as thermal conduction progressed. During the final part of the treatment the increase in thermal lesion volume was caused mainly by thermal conduction, rather than absorption of electrical energy.

**Table 3:** Ablation data from the computer simulation protocols

Case description		V_in_(V)	I(A)	Z(Ω)	Power (W)
1	F	37.1	0.099	373	3.686627
2	F	28.8	0.144	200	4.142817
3	FT	29	0.109	267	3.150734
4	FT	22.7	0.147	155	3.333956
5	FT	22.8	0.163	140	3.71184
6	FT	23	0.134	171	3.07717
7	FT	22.2	0.193	115	4.301216
8	FT	23.6	0.120	196	2.840624
9	F	60–39	-	-	-
10	FT	60–32	-	-	-

F = geometry with only fatty tissue, FT = geometry with fatty tissue and embedded tumour. For cases using temperature dependent conductivity the presented current, impedance and power are the final values at the end of the treatment.

## Discussion

### Comments on the FEM results

The largest thermal lesions were created using temperature dependent electrical conductivity and steady state solving. Furthermore, temperature dependent electrical conductivity mimics the impedance behaviour in the in vitro studies, which demonstrated a decreasing impedance of up to 50%. Hence, only temperature dependent electrical conductivity was used to document the effect of tumour spatial shape and the influence of time dependence. The baseline impedance in the experimental studies varied between 80 and 200 Ω. During FEM simulation the baseline impedance varied between 51 and 373 Ω, using only tumour tissue or only fat tissue, respectively. Consequently, the electrical parameters in the in vitro experiments and in the FEM model correspond well. In the models with only fat tissue, the heat was shifted towards the tip and away from the electrode because of the cooling along the electrode shaft and the increased SAR created by the curvature of the electrode tip. The shift in temperature between corresponding models with and without tumour is primarily explained by the shift in absorbed electrical power density (figures 7 and 9). In the models with tumour, the electrical absorption was relatively higher in the tumour tissue and relatively lower in the fat tissue compared to a non-tumour model. Even though the thermal lesion volume decreased during steady state simulation, preferential heating of the tumour increased. The thermal lesion volume decreased because of the shorter treatment time, i.e. less energy is transmitted by thermal conduction to heat the distant regions. Initially, the tumour shape could be detected in the isothermal lines. but with time this shape effect gradually decreased. Hence, as entropy increased and thermal conduction levelled out the differences in temperature with time, the temperature difference between the tumour and the fat gradually decreased to the steady state. The width of the tumour, compared to the length, has a more pronounced effect on the preferential heating. With increasing tumour width, both the location of the temperature maximum and the thermal lesion margin are pushed away from the electrode, along the symmetry axis, In case 8, the thermal lesion margin is closer to the electrode than in the original fat model. Yet the region near the electrode is relatively hotter in case 8. It seems that the heat dissipation from thin tumours decrease the lesion size. However, during transient analysis, this effect might decrease. The altering of the tumour length only changed the tumour in the region where the contribution to the impedance and resistive heating were small. Hence, almost no dependence on tumour length is detected. The tissue near the electrode is heated mainly by the absorbed electrical energy, while regions further away are mainly heated by thermal conduction. Thus, the preferential heating of the tumour is governed by the electrical parameters near the electrode, whereas thermal parameters become increasingly important further away.

The effects of temperature dependent electrical conductivity during RFA have been studied by Chang [18]. Temperature dependent conductivity results in increased current density, whereas the electric field remains almost constant compared to constant conductivity. The maximum temperature increases by 5–8%, using constant voltage between the electrodes. Furthermore, one study has investigated the preferential heating of hepatic tumours [35]. The study concludes that ablation at low frequencies, where the difference in electrical parameters are relatively higher, may preferentially target tumour tissue. This effect only arises if the active electrode is in contact with both liver and tumour tissue. The extent of the effect is also dependent on probe geometry and control algorithm. Moreover, Tungjitkusolmun, et al. have investigated the sensitivity of tissue parameters on thermal lesions for radio frequency cardiac ablation [22]. Their study shows that the accuracy of tissue property values is critical to FEM modelling.

### Limitations of the computer model

We have limited the computer model to include two tissue types: fat and tumour, giving a fair representation of the real situation. The electrical properties of glandular and connective tissue are similar to those of cancer. Thus, connective and glandular tissue are also preferentially heated by the RF-energy, which was confirmed in the in vitro studies. Hence, incorporation of glandular and connective tissue provides little additional information on the preferential heating of the tumour. To correctly mimic the SAR situation during RF ablation, a three dimensional model must be used, i.e. the iso-potential surface must increase with the distance squared from the electrode. This is for obvious reasons not the case in two dimensional or one dimensional Cartesian models. However, if the situation demonstrates rotational symmetry a two dimensional axially symmetric model can be used instead to significantly decrease calculation time and related computer resources. Non-linear models, transient analysis, and the use of different tissue types made rotational symmetry essential in our case. The tumour shapes considered are not claimed to be perfect representations of the true situation. A better representation would be a core tumour from which thin outgrowths extend. However, this configuration is impossible to achieve with rotational symmetry and with current limitations in our computational resources. Additionally, such a model would have too many variables to easily assess any conclusions. In this study the tumours introduced were shaped to present a similar situation as in the tumour strands extending from the core. We have, as a first step, developed a model with rotational symmetry and only two variables, thickness and length, which can demonstrate possible preferential heating of the tumour. Potential effects of temperature dependent thermal conductivity are not incorporated in this model. This phenomenon is usually not accounted for because of its low temperature coefficient, approximately 0.1%/°C for muscle tissue [36], and no breast specific data are available. The change in tissue electrical conductivity during heating has reversible and permanent effects [37]. The permanent temperature effect increases with time and appears to be the result of structural changes of the tissue. Our model only accounts for the reversible part of the temperature dependence. However, the permanent temperature effect is less significant compared to the reversible effect. Furthermore, the 2%/°C coefficient used in this study is an over estimation of the measured reversible part, giving a fair approximation of the total change during our time interval of heating.

Regarding the FEM simulations, a strict error analysis is not considered possible for this multi-field problem. An indication of accuracy in the formulation is the conservationof energy introduced by the source. This was checked and found highly reliable. This finding verified that the element approximation is able, in the limit, to reproduce the governing mathematical formulation. With respect to grading of the element mesh, a Cauchy convergence test was used to assess if the mesh had an appropriate size. The accuracy level in the two aspects of the simulations is thereby considerably higher than for the tissue material parameters used. The computer and experimental studies were performed in an in vitro situation, disregarding possible effects of blood perfusion. Thus, the lesion size is overestimated compared to the in vivo case. Furthermore, the preferential heating of the tumour is probably decreased due to the temperature dependent blood flow.

### Comparison between experimental results and computer model

We have shown that RFA of breast cancer in vitro results in preferential heating of the tumour during both the experimental and the computer simulation studies. However, during computer simulation of thin tumours, the preferential heating at the thermal lesion margin vanishes due to increased heat conduction from the tumour. Thus, the preferential heating of the tumour observed in the experimental studies was more pronounced, especially in the long outgrowths extending from the core tumour, indicating that additional effects other than just differences in electrical and thermal parameters must be involved. During cancer growth, fibrous septa membranes are produced by the tumour and existing membranes are pushed in front of the tumour, creating numerous thin membrane layers at the tumour interface. These septa layers between the cancer tissue and the fatty tissue could have an additional electrical or thermal insulating effect. Thermal insulating membranes would probably increase the temperature in the tumour in distant regions where they dominate.

## Authors' contributions

HW contributed with the initial idea that dissimilar tissue properties might result in preferential heating of the tumour. He also filed the ethical applications and performed the RF-ablation for the in vitro study with VE. VE initiated the study to investigate if dissimilar tissue parameters could invoke preferential heating of the tumour by FEM analysis, performed all FEM analysis and summoned the results in this paper. IS performed the mastectomies for the in vitro study and summarized the results for this study. BS advised on how to model a tumour i.e. shape, composition and location, etc. He also performed the histological investigation in the in vitro study. SR assisted on the general concept. AE advised and helped on many matters during the FEM modelling process. Additionally AE helped with the proofreading process and provided the needed facilities. All authors read and approved the final manuscript.

## References

[B1] Solin LJ, Fourquet A, Vicini FA, Taylor M, Olivotto IA, Haffty B, Strom EA, Pierce LJ, Marks LB, Bartelink H, McNeese MD, Jhingran A, Wai E, Bijker N, Campana F, Hwang WT (2005). Long-term outcome after breast-conservation treatment with radiation for mammographically detected ductal carcinoma in situ of the breast. Cancer.

[B2] Kricker A, Armstrong B (2004). Surgery and outcomes of ductal carcinoma in situ of the breast: a population-based study in Australia. Eur J Cancer.

[B3] Bradley CJ, Given C, Baser O, Gardiner J (2003). Influence of surgical and treatment choices on the cost of breast cancer care. Eur J Health Econ.

[B4] Given C, Bradley C, Luca A, Given B, Osuch JR (2001). Observation interval for evaluating the costs of surgical interventions for older women with a new diagnosis of breast cancer. Med Care.

[B5] Singletary S (2001). Minimally invasive techniques in breast cancer treatment. Semin Surg Oncol.

[B6] Hall-Craggs MA, Vaidya JS (2002). Minimally invasive therapy for the treatment of breast tumours. Eur J Radiol.

[B7] Gazelle GS, Goldberg SN, Solbiati L, Livraghi T (2000). Tumor ablation with radio-frequency energy. Radiology.

[B8] Noguchi M (2003). Minimally invasive surgery for small breast cancer. J Surg Oncol.

[B9] Singletary ES (2003). Feasibility of radiofrequency ablation for primary breast cancer. Breast Cancer.

[B10] Ekstrand V (2002). Development and Clinical Evaluation of Therapeutic Techniques based on Acoustic and Electromagnetic Energy. Licentiate thesis.

[B11] Izzo F, Thomas R, Delrio P, Rinaldo M, Vallone P, DeChiara A, Botti G, D'Aiuto G, Cortino P, Curley SA (2001). Radiofrequency ablation in patients with primary breast carcinoma. Cancer.

[B12] Livraghi T, Goldberg N, Monti F, Bizzini A, Lazzaroni S, Meloni F, Pellicano S, Solbiati L, Gazelle S (1997). Saline-enhanced Radio Frequency Tissue Ablation in the Treatment of Liver Metastases. Radiology.

[B13] Goldberg S, Gazelle S, Solbiati L, Rittman W, Mueller P (1996). Radiofrequency Tissue Ablation: Increased Lesion Diameter with a Perfusion Electrode. Acad Radiol.

[B14] Burak WE, Agnese DM, Povoski SP, Yanssens TL, Bloom KJ, Wakely PE, Spigos DG (2003). Radiofrequency ablation of invasive breast carcinoma followed by delayed surgical excision. Cancer.

[B15] Izzo F, Thomas R, Delrio P, Rinaldo M, Vallone P, DeChiara A, Botti G, D'Aiuto G, Cortino P, Curley SA (2001). Radiofrequency ablation in patients with primary breast carcinoma: a pilot study in 26 patients. Cancer.

[B16] Hayashi AH, Silver SF, van der Westhuizen NG, Donald JC, Parker C, Fraser S, Ross AC, Olivotto IA (2003). Treatment of invasive breast carcinoma with ultrasound-guided radiofrequency ablation. Am J Surg.

[B17] Fornage BD, Sneige N, Ross MI, Mirza AN, Kuerer HM, Edeiken BS, Ames FC, Newman LA, Babiera GV, Singletary SE (2004). Small (< or = 2-cm) breast cancer treated with US-guided radiofrequency ablation: feasibility study. Radiology.

[B18] Chang I (2003). Finite Element Analysis of Hepatic Radiofrequency Ablation Probes using Temperature-Dependent Electrical Conductivity. Biomed Eng Online.

[B19] Haemmerich D, Tungjitkusolmun S, Staelin ST, Lee FT, Mahvi DM, Webster JG (2002). Finite-element analysis of hepatic multiple probe radio-frequency ablation. IEEE Trans Biomed Eng.

[B20] Tungjitkusolmun S, Staelin ST, Haemmerich D, Tsai JZ, Webster JG, Lee FT, Mahvi DM, Vorperian VR (2002). Three-Dimensional finite-element analyses for radio-frequency hepatic tumor ablation. IEEE Trans Biomed Eng.

[B21] Haemmerich D, Chachati L, Wright AS, Mahvi DM, Lee FT, Webster JG (2003). Hepatic radiofrequency ablation with internally cooled probes: effect of coolant temperature on lesion size. IEEE Trans Biomed Eng.

[B22] Tungjitkusolmun S, Woo EJ, Cao H, Tsai JZ, Vorperian VR, Webster JG (2000). Thermal – electrical finite element modelling for radio frequency cardiac ablation: effects of changes in myocardial properties. Med Biol Eng Comput.

[B23] Osman MM, Afify EM (1988). Thermal modeling of the malignant woman's breast. J Biomech Eng.

[B24] Sudharsan NM, Ng EY, Teh SL (1999). Surface Temperature Distribution of a Breast With and Without Tumour. Comput Methods Biomech Biomed Engin.

[B25] Gautherie M, Qenneville Y, Gros CH (1975). Thermogenesis of mammary epitheliomas. III. Study, by means of fluvography, of the thermal conductivity of mammary tissue and of the influence of tumor vascularization. Biomedicine.

[B26] Erdmann B, Lang J, Seebass M (1998). Optimization of temperature distributions for regional hyperthermia based on a nonlinear heat transfer model. Ann N Y Acad Sci.

[B27] Werner J, Buse M (1988). Temperature profiles with respect to inhomogeneity and geometry of the human body. J Appl Physiol.

[B28] Gautherie M (1980). Thermopathology of breast cancer: measurement and analysis of in vivo temperature and blood flow. Ann N Y Acad Sci.

[B29] Jossinet J (1996). Variability of impedivity in normal and pathological breast tissue. Med Biol Eng Comput.

[B30] Grimnes S, Martinsen OG (2000). Bioimpedance & Bioelectricity.

[B31] Foster KR, Schwan HP (1989). Dielectric properties of tissues and biological materials: A critical review. Crit Rev Biomed Eng.

[B32] Cook RD, Malkus DS, Plesha ME, Witt RJ (2002). Concepts and Applications of Finite Element Analysis.

[B33] Zienkiewicz OC, Taylor RL (2000). The Finite Element Method. The basis.

[B34] Graham SJ, Chen L, Leitch M, Peters RD, Bronskill MJ, Foster FS, Henkelman RM, Plewes DB (1999). Quantifying tissue damage due to focused ultrasound heating observed by MRI. Magn Reson Med.

[B35] Haemmerich D, Mahvi DM, Lee FT, Webster JG (2002). RF ablation at audio frequencies preferentially targets tumor – A Finite Element Study. Proceedings EMBS-BMES Houston.

[B36] Tungjitkusolmun S, Cao H, Tsai JZ, Webster JG (1997). Using ANSYS for three-dimensional electrical-thermal models for radio-frequency catheter ablation. Proceedings – 19th International Conference IEEE/EMBS: 30 October – 2 November 1997; Chicago.

[B37] Pop M, Molckovsky A, Chin L, Kolios MC, Jewett MA, Sherar MD (2003). Changes in dielectric properties at 460 kHz of kidney and fat during heating: importance for radio-frequency thermal therapy. Phys Med Biol.

